# *CDKN2B* expression and subcutaneous adipose tissue expandability: Possible influence of the 9p21 atherosclerosis locus

**DOI:** 10.1016/j.bbrc.2014.03.075

**Published:** 2014-04-18

**Authors:** Per-Arne Svensson, Björn Wahlstrand, Maja Olsson, Philippe Froguel, Mario Falchi, Richard N. Bergman, Philip G. McTernan, Thomas Hedner, Lena M.S. Carlsson, Peter Jacobson

**Affiliations:** a Institute of Medicine, The Sahlgrenska Academy at University of Gothenburg, Sweden; b Department of Genomics of Common Disease, School of Public Health, Imperial College London, UK; c Diabetes and Obesity Research Institute, Cedars-Sinai Medical Center, Los Angeles, CA, USA; d Division of Metabolic and Vascular Health, Warwick Medical School, University of Warwick, Coventry, UK

**Keywords:** CVD, cardiovascular disease, SAT, subcutaneous adipose tissue, VAT, visceral adipose tissue, CDKN2A/B, cyclin-dependent kinase inhibitor 2A/B, ANRIL, antisense noncoding RNA in the INK4 locus, MTAP, methylthioadenosine phosphorylase, BMI, body mass index, TAG, triacylglycerol, CDKN2B, Adipose tissue, Chromosome 9p21

## Abstract

Risk alleles within a gene desert at the 9p21 locus constitute the most prevalent genetic determinant of cardiovascular disease. Previous research has demonstrated that 9p21 risk variants influence gene expression in vascular tissues, yet the biological mechanisms by which this would mediate atherosclerosis merits further investigation. To investigate possible influences of this locus on other tissues, we explored expression patterns of 9p21-regulated genes in a panel of multiple human tissues and found that the tumor suppressor *CDKN2B* was highly expressed in subcutaneous adipose tissue (SAT). *CDKN2B* expression was regulated by obesity status, and this effect was stronger in carriers of 9p21 risk alleles. Covariation between expression of *CDKN2B* and genes implemented in adipogenesis was consistent with an inhibitory effect of *CDKN2B* on SAT proliferation. Moreover, studies of postprandial triacylglycerol clearance indicated that *CDKN2B* is involved in down-regulation of SAT fatty acid trafficking. *CDKN2B* expression in SAT correlated with indicators of ectopic fat accumulation, including markers of hepatic steatosis. Among genes regulated by 9p21 risk variants, *CDKN2B* appears to play a significant role in the regulation of SAT expandability, which is a strong determinant of lipotoxicity and therefore might contribute to the development of atherosclerosis.

## Highlights

• The tumor suppressor gene *CDKN2B* is highly expressed in human adipose tissue.
• Risk alleles at the 9p21 locus modify *CDKN2B* expression in a BMI-dependent fashion.
• There is an inverse relationship between expression of *CDKN2B* and adipogenic genes.
• *CDKN2B* expression influences to postprandial triacylglycerol clearance.
• *CDKN2B* expression in adipose tissue is linked to markers of hepatic steatosis.

## 1 Introduction

Manifestations of atherosclerosis such as myocardial infarction and stroke are leading causes of death worldwide [1]. Beside environmental factors including lifestyle, the risk of cardiovascular disease (CVD) is also influenced by genetic variation, and several genomic regions have been linked to CVD [2]. The strongest of these is located on chromosome 9p21, where single-nucleotide polymorphisms (SNPs) located within a 53-kilobase interval have shown consistent and strong association with CVD [3–7].

The 9p21 locus confers CVD risk through a mechanism which appears to be independent from conventional risk factors, including dyslipidaemia, hypertension, and impaired glucose metabolism [5,8–10]. However, a recent study indicated that the effect of risk variants is modified by diet [11].

Most of the CVD-associated SNPs within the 9p21 locus are located in a so-called gene desert – a genomic region devoid of protein-coding genes. Recent studies of gene expression in vascular tissues or peripheral mononuclear blood cells demonstrate that the 9p21 disease variants may be involved in the regulation of nearby genes, including cyclin-dependent kinase inhibitor 2A/B (*CDKN2A*), *CDKN2B* and antisense noncoding RNA in the *INK4* locus (*ANRIL*) [12–17]. However, the reported associations between risk variants and gene expression appear somewhat conflicting, as reviewed by Cunnington & Keavney [18]. One reason for this may be that the CVD risk SNPs affect the expression of *ANRIL*, which in turn may affect expression of other nearby genes via epigenetic mechanisms [19].

Although a regulatory role of the 9p21 locus on gene expression in vascular tissues has been found, the biological mechanisms that would link this to human atherosclerosis development are still not fully elucidated. Studies in humans and in mice models have shown that vascular mechanisms, such as smooth muscle cell proliferation [20], response to inflammatory signaling [21] and macrophage phagocytosis [22], may be involved. However, the CVD-associated variants at the 9p21 locus may affect the expression of adjacent genes also in extravascular tissues. We therefore explored the possible regulatory influences of this locus in human tissues not previously studied in this context and subsequently assessed the findings in carefully phenotyped cohorts.

## 2 Materials and methods

All subjects gave written informed consent prior to inclusion in the study. The studies were conducted in accordance with the Declaration of Helsinki and were approved by the local ethics committees.

### 2.1 Study populations

The Sibpair study includes 154 Swedish nuclear families recruited via BMI-discordant adult sibling pairs (BMI difference ⩾10 kg/m^2^) as described previously [23]. Subjects underwent extensive phenotypic examinations. In the current analysis, gene expression data were available for 354 adult sibs (106 males) from 151 families. BMI ranged from 16.9 to 57.5 kg/m^2^ (Supplementary Table II).

Lean and obese women from UK undergoing elective surgery were recruited and abdominal subcutaneous and omental adipose tissue biopsies were obtained [24] (Supplementary Table II).

Obese Swedish subjects underwent 18 weeks of caloric restriction by means of very-low calorie diets [25]. Subcutaneous adipose tissue biopsies were taken at baseline, week 8, and week 18 from a subset of 10 subjects (Supplementary Table II).

### 2.2 DNA microarray analysis

Adipose tissue RNA preparation and microarray analysis (U133 plus 2.0 microarrays, Affymetrix, Santa Clara, CA, USA) were performed as previously described [26].

### 2.3 Real-time PCR analysis

RNA was reverse transcribed using the High Capacity cDNA RT kit (Applied Biosystems, Foster City, CA, USA). Reagents for real-time PCR analysis of *CDKN2B* (Hs00793225_m1), *LPR10* (Hs00204094_m1), and *PPIA* (Hs99999904_m1) were purchased from Applied Biosystems and used according to the manufacturer’s protocol.

### 2.4 Genotyping

DNA was isolated from whole blood and genotyping of the rs10757278 and rs2383207 was performed using the TaqMan-based system (Applied Biosystems) and assays (C_11841860_10 and C_15789010_10). Successful genotypes were obtained from 95.2% for rs10757278 and 96.7% for rs2383207.

### 2.5 Body composition

A subset (*n* = 137) from the Sibpair study underwent computed tomography measurements of body composition. Visceral and subcutaneous fat were estimated from a scan at the level of the iliac crest. Leg subcutaneous and intermuscular fat were estimated at the mid-thigh level. Tissue areas were determined as previously described [27].

### 2.6 Intravenous glucose tolerance test and biochemistry

Frequent-sampling intravenous glucose tolerance test was performed in the Sibpair cohort for the assessment of insulin sensitivity (SI) and disposition index (DI), and blood biochemistry was performed as previously described [26].

### 2.7 Postprandial lipid metabolism

Postprandial triacylglycerol (TAG) clearance in subjects of the Sibpair study was measured in serum drawn in the fasting state and hourly for seven hours after ingestion of a standard meal composed of 41.3 g protein (22% of energy content), 46.3 g fat (54%), and 46.8 g carbohydrates (25%), corresponding to 769 kcal. Clearance was expressed as area under the curve, adjusted for fasting value.

### 2.8 Statistical analysis

Statistical analyses were performed using the SAS software package (v. 9.1.3, SAS Institute Inc, Cary, NC, USA). Quantitative data were transformed towards normal distribution using Box-Cox power transformations. Outliers beyond 3 standard deviations from the trait mean were excluded. Additional statistical analyses are described in the Method supplement.

## 3 Results

### 3.1 *CDKN2B* is highly expressed in adipocytes

**Fig. 1:**
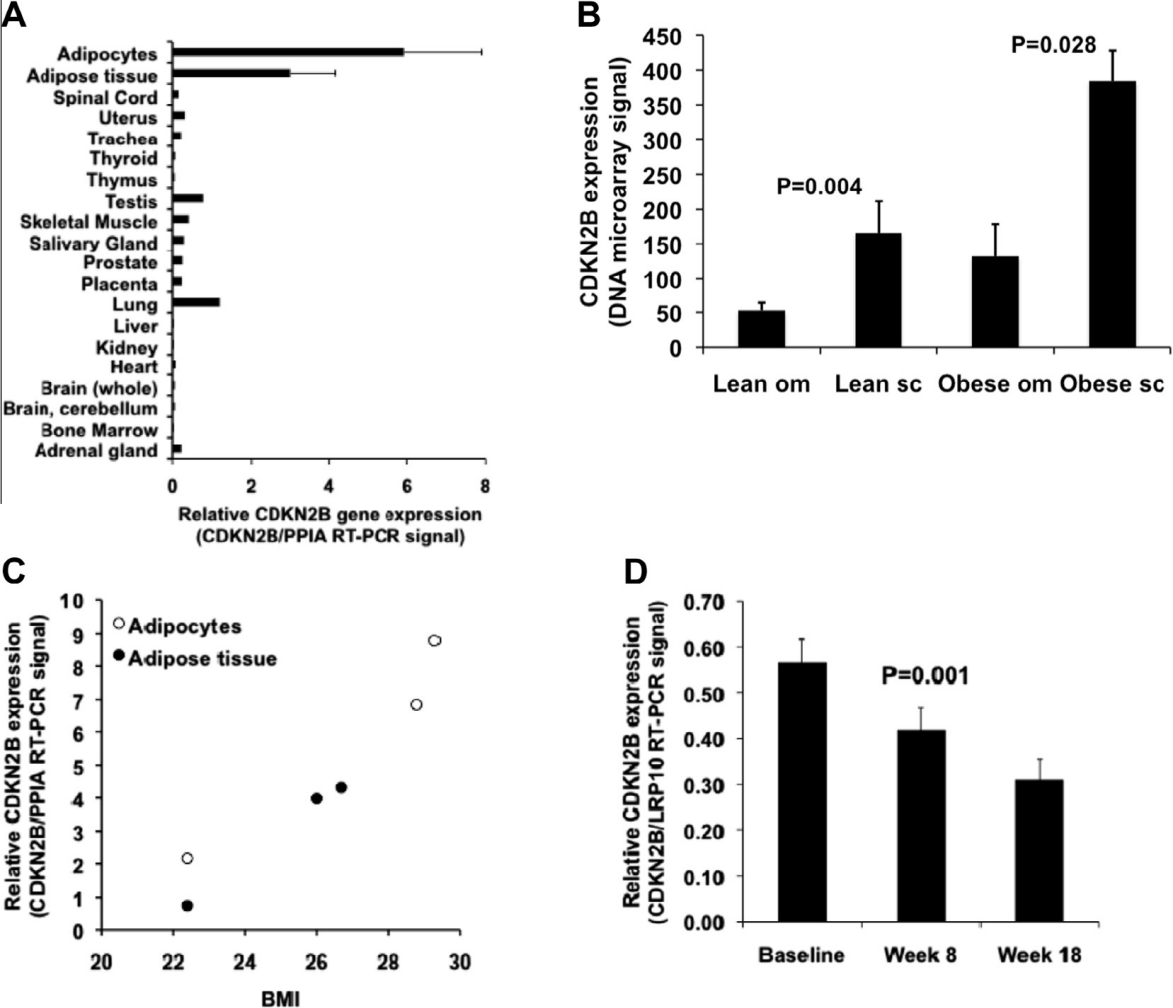
Expression of *CDKN2B* in multiple human tissues and covariation of *CDKN2B* expression in subcutaneous adipose tissue with energy balance. (A) *CDKN2B* expression analyzed by RT-PCR in adipocytes (*n* = 3) and adipose tissue (*n* = 3) from healthy volunteers together with 18 other human tissues from the Human Total RNA Master Panel II. *CDKN2B* expression was normalized to reference gene *PPIA*. Values are mean ± SEM, when applicable. (B) Comparison of *CDKN2B* expression, analyzed by DNA microarray, between omental (om) and subcutaneous (sc) adipose tissue among lean and obese subjects. *P* values are from paired *T* tests of differences between fat depots. (C) Relationship between BMI and *CDKN2B* expression analyzed by RT-PCR in subcutaneous adipose tissue at the whole-tissue level and specifically in adipocytes from healthy volunteers. The measurements are the same as in panel A. *CDKN2B* expression was normalized to reference gene *PPIA*. (D) Response in subcutaneous adipose tissue of *CDKN2B* expression, analyzed by RT-PCR, to 8 and 18 weeks of caloric restriction in obese adults. *CDKN2B* expression was normalized to reference gene *LRP10*. *P* value is for repeated measures ANOVA from a linear mixed model.

The expression of 9p21 positional candidate genes *ANRIL*, *CDKN2A*, *CDKN2B*, and *MTAP* were analyzed by microarray in a panel of 65 human tissues. The *CDKN2B* gene showed the highest expression in subcutaneous adipose tissue (SAT) whereas the other positional candidate genes displayed low signals in all tissues investigated (Fig. S1). The tissue distribution pattern of *CDKN2B* expression was also investigated by real-time PCR in a subset of tissues and cells. In this analysis, the highest expression of CDKN2B was observed in subcutaneous adipocytes (Fig. 1A). In view of the association between obesity and CVD, which is independent of conventional CVD risk factors [28], we pursued the investigation of *CDKN2B* expression in adipose tissue further.

### 3.2 Adipose tissue *CDKN2B* expression varies among fat depots and is regulated by energy balance

Given that different adipose tissue depots have varying influences on the metabolism [29], *CDKN2B* expression was investigated in paired SAT and omental adipose tissue biopsies (*n* = 10) by microarray. Higher expression in SAT compared to omental adipose tissue was found in both lean (3.2-fold, *P* = 0.004) and obese (2.9-fold, *P* = 0.028) subjects (Fig. 1B).

**Fig. 2:**
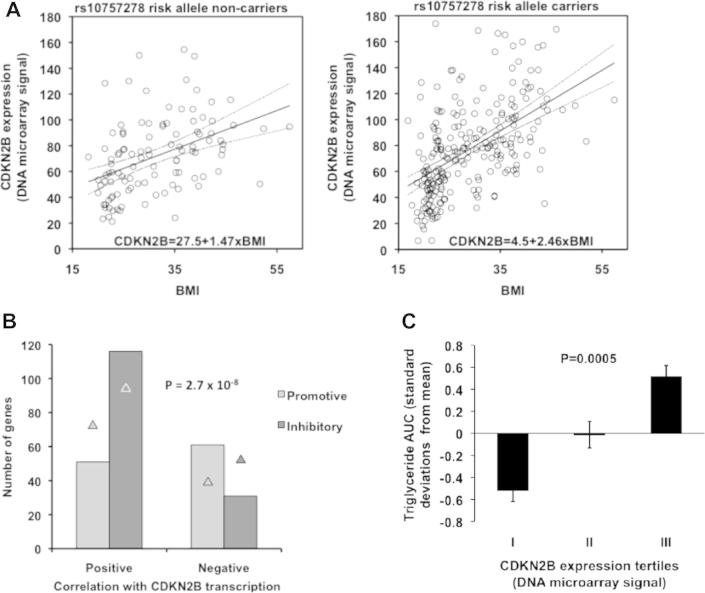
Covariation of *CDKN2B* expression in subcutaneous adipose tissue with cardiovascular risk alleles, adipose tissue regulatory genes, and postprandial lipid clearance. (A) Data from the Sibpair study, showing the relationship between BMI and *CDKN2B* expression (microarray) in SAT from carriers of the rs10757278 risk allele (right) and non-carriers (left), where the slope of the regression line is steeper for risk allele carriers compared to non-carriers (*P* < 0.05 for genotype-by-*CDKN2B* interaction). Dotted curves indicate the 95-percent confidence intervals for the regression lines. (B) Association analysis, in the Sibpair study, of 259 adipose transcripts analyzed by microarray with promotive or inhibitory effects on tissue growth vs. their positive or negative correlation with *CDKN2B* expression. Triangles indicate expected counts under the null hypothesis of no association. The *P* value denotes the significance of a *X*^2^ test (*X*^2^ = 30.9; df = 1). (C) Data from the Sibpair study, showing lipid clearance from serum during 7 h after a standardized meal (*n* = 225). Data are expressed as area under the curve (AUC) for postprandial serum triacylglycerol, categorized by *CDKN2B* expression tertile. Data are represented as standard deviations from the overall mean. Error bars are ±standard errors. *P* value is for the effect of *CDKN2B* tertile on triacylglycerol AUC, adjusted for fasting value, BMI, and non-independence among siblings as assessed by a linear mixed model.

A correlation between BMI and SAT *CDKN2B* expression was found in the Sibpair study (*r* = 0.46, *P* = 6.3 × 10^−20^) (see Fig. 2A). This finding was confirmed by real-time PCR in a small set of SAT and subcutaneous adipocyte samples (Fig. 1C).

Next, we investigated whether *CDKN2B* expression in SAT is regulated by weight loss following 18 weeks of severe caloric restriction in ten obese individuals. A mean weight loss of 28.4 (range 21.0–38.6) kilograms induced decreased *CDKN2B* expression (Fig. 1D, *P* = 0.001).

### 3.3 BMI-dependent association between 9p21 variants and adipose tissue *CDKN2B* expression

CVD risk alleles at 9p21 locus have been shown to influence *CDKN2B* expression and therefore the associations between risk alleles of the rs10757278 SNP and *CDKN2B* expression in SAT were investigated in the Sibpair study. No direct association between expression and risk allele carrier status was found. However, in a model which adjusted for non-independence among relatives, sex, age and BMI, a positive correlation between risk allele carrier status and *CDKN2B* expression was found (*P* = 0.002). Moreover, risk variant carrier status modified the previously described association between BMI and *CDKN2B* expression, yielding a steeper slope in carriers of the risk allele, compared with non-carriers (Fig. 2A) (*P* = 0.043 for rs10757278-by-BMI interaction). Similar results were obtained for the risk allele (G) of rs2383207, both for the main effect (*P* = 0.0052) and for the interaction (*P* = 0.048).

### 3.4 Inverse relationship between expression of *CDKN2B* and genes implicated in adipose tissue expandability

The known anti-proliferative effects of *CDKN2B* [30], its high expression in SAT and its link to obesity suggest that this gene is involved in the regulation of SAT growth and expandability in response to changes in energy balance. We sought further support for this theory using the Sibpair study to examine expression covariation between *CDKN2B* and genes implemented in various aspects of adipose tissue expandability, such as adipogenesis, proliferation and angiogenesis.

Among the genes whose expression correlated significantly with *CDKN2B* expression, 259 genes could be classified as having promotive or inhibitory effects on adipose tissue expandability (see Supplementary methods and Supplementary Table I). Aiming to discern a global effect of *CDKN2B* on these 259 genes, we performed an association analysis between the promotive or inhibitory effects of the genes versus their positive or negative correlation with *CDKN2B* expression. As shown in Fig. 2B, the majority of promotive genes correlated negatively with *CDKN2B*, whereas the majority of inhibitory genes correlated positively with *CDKN2B*. Consistently, there were lower than expected frequencies of promotive genes positively correlated and inhibitory genes negatively correlated with *CDKN2B* expression (*X*^2^ = 30.9, *P* = 2.7 × 10^−8^).

The classical initiator of adipogenesis, peroxisome proliferator-activated receptor gamma [31], was negatively correlated with *CDKN2B*, whereas adipogenesis inhibitors such as Cyclin D1 [32] and WW domain containing transcription regulator 1 [33] were positively correlated with *CDKN2B*.

Angiogenesis is essential to adipose tissue growth, and 35 angiogenesis genes were highly expressed in SAT and significantly correlated with *CDKN2B* expression. Among antiangiogenic genes, thrombospondin 1, caveolin 1 and 2 were positively correlated with *CDKN2B*. Expression of important proangiogenic genes e.g., angiogenin and vascular endothelial growth factor A, were negatively correlated with *CDKN2B* expression (Supplementary Table I).

### 3.5 Increased CDKN2B expression in SAT impedes postprandial lipid clearance

Impairment of adipose tissue growth and lipid accommodation manifests itself as ectopic lipid accumulation. Therefore, we investigated covariation of *CDKN2B* expression with clinical indicators of a “lipodystrophic” phenotype.

One acute effect of such impairment, which can be studied in the short term, is reduced clearance from serum of lipids derived from the consumption of a meal. We measured postprandial triacylglycerol (TAG) levels following a standardized meal in subjects from the Sibpair study, subdivided into tertiles of SAT *CDKN2B* expression. The results show that high adipose tissue CDKN2B expression is linked to high postprandial TAG levels (Fig. 2C, *P* = 0.0005). The effect of *CDKN2B* expression level was independent of BMI, which is a strong determinant of postprandial lipidaemia.

### 3.6 *CDKN2B* expression in SAT and ectopic lipid accumulation

Other indicators of lipodystrophy or lipotoxicity include the abundance of visceral, intramuscular and hepatic fat, insulin resistance, and impaired pancreatic beta cell function.

In a subset of the Sibpair cohort, we used computed tomography to measure body composition at the iliac crest and mid-thigh levels. These levels were chosen to obtain estimates of abdominal subcutaneous *vs.* visceral adipose tissue (VAT) and extremity subcutaneous vs. intermuscular adipose tissue, respectively. *CDKN2B* expression was positively correlated with the majority of these adipose tissue measurements (Table 1, model 1). Moreover, the VAT/SAT ratio and the amount of SAT in the thigh correlated significantly to *CDKN2B* expression even after adjustment for BMI (Table 1, model 2).

Using serum alanine aminotransferase (S-ALAT) as a marker [34], we found evidence of a relationship between *CDKN2B* expression in SAT and hepatic steatosis, since S-ALAT was positively correlated with *CDKN2B* expression, also after BMI adjustment.

## 4 Discussion

The mechanism that links regulatory sequence variants within the 9p21 gene desert with CVD has in recent years been intensely investigated. Studies have suggested mechanisms in vascular tissue that may contribute to this link [20–22]. Our study suggests a new possible route how the 9p21 locus may affect CVD development via alteration in adipose tissue *CDKN2B* expression.

We found that *CDKN2B* is highly expressed in SAT, and its expression shows covariation with energy balance (higher expression in obese subjects and down-regulated expression during caloric restriction-induced weight-loss). Considering the capability of adipose tissue to expand and retract throughout life, it is not surprising that a gene implemented in cell cycle regulation is variably expressed in this tissue. In line with this, we observed a correlation pattern between *CDKN2B* expression and expression of genes involved in adipose tissue expandability, suggesting that CDKN2B has an inhibitory effect on adipogenesis and SAT expandability. A recent study by Horswell et al. has provided direct experimental support to the hypothesis that CDKN2B is important for adipogenesis. They show that knock-down of Cdkn2b expression in a mouse adipocyte cell line results in increased adipogenesis [35]. Moreover, we found substantially higher *CDKN2B* expression in subcutaneous than in visceral adipose tissue, suggesting a larger restraint on the proliferative capacity and adipogenesis of the former compared to the latter, which might contribute to their altered relative abundances.

Inadequate SAT expandability and fat storage capacity is the disease-causing mechanism behind lipodystrophy syndromes. It has been proposed that the dysmetabolic consequences of severe obesity are partly attributable to “acquired lipodystrophy” which develops as the adipocytes reach their maximal capacity to accommodate dietary lipids, causing lipid accumulation in visceral fat depots and non-adipose tissues [36]. McQuaid et al. observed that obese men, despite substantially greater adipose tissue mass, showed depressed adipose tissue fat storage capacity compared with lean controls [37].

Several studies have investigated the link between CVD risk SNPs at the 9p21 locus and expression of *CDKN2B* in vascular tissue and leukocytes. However, the results from these studies are conflicting [18]. This may partially be due to the risk variant influence on the expression of *ANRIL*, which in turn may affect *CDKN2B* expression via epigenetic mechanisms [19]. In addition, confounders such as age and BMI were not always accounted for. We found a BMI dependent association between risk allele carrier status and CDKN2B expression in adipose tissue. More importantly, the risk variants modified the correlation between BMI and *CDKN2B* expression, suggesting that the expandability of SAT in risk allele carriers is reduced. Visel et al. [38] showed that deletion of a 70-kilobase noncoding region on mouse chromosome 4, orthologous to the human 9p21 CVD risk interval resulted in reduced *Cdkn2a* and *Cdkn2b* expression. Compared to the wild type, a faster weight gain was seen in mice lacking this region, whereas a difference in aortic fatty-lesions after high fat diet could not be shown. In view of our findings, the results may be interpreted in terms of reduced lipotoxicity due to loss of *Cdkn2b*-mediated inhibition of SAT. However, a recent study showed that *Cdkn2b* deficient mice on an ApoE deficient background develop more atherosclerosis compared to ApoE deficient mice with an intact *Cdkn2b* gene [22].

Our study lacked statistical power to assess direct associations between CVD risk alleles and lipotoxicity phenotypes. A large study on two independent human cohorts, showed significant interaction between unhealthy diet and 9p21 risk alleles on CVD, whereas carrier status did not affect risk in subjects on healthier diet [11]. Those results are consistent with ours, which showed higher postprandial TAG in subjects with high *CDKN2B* expression, which in turn was higher in 9p21 risk allele carriers. Relevant to our findings and their proposed contribution to CVD risk, postprandial lipid dysmetabolism has been shown to independently predict CVD [39–41]. The reason that the CVD risk of the 9p21 locus have been shown to be independent of dyslipidaemia may be due to that only fasting blood lipid levels have been investigated.

Based on our findings, we propose that *CDKN2B* is involved in the regulation of SAT in response to changes in energy balance. This study suggests that 9p21 risk alleles contribute to CVD risk indirectly via inhibition of adipogenesis and adipose tissue expandability which may promote ectopic fat accumulation.

## Figures and Tables

**Table 1:** Correlations of subcutaneous adipose tissue (SAT) *CDKN2B* expression with body-composition and metabolic traits susceptible to the effects of ectopic lipid accumulation or suppressed SAT lipid accommodation.

Phenotype	Model 1		Model 2	
	*r*	*P*-Value	*r*	*P*-Value
Computed tomography areas at iliac crest level^a^				
Visceral adipose tissue	0.33	0.0003	0.07	0.472
Subcutaneous adipose tissue	0.24	0.028	−0.06	0.500
VAT/SAT	0.19	0.007	0.26	0.002

Computed tomography areas at mid-thigh level^a^				
Intermuscular adipose tissue	0.33	2.2 × 10^−6^	−0.00	0.974
Subcutaneous adipose tissue	0.13	0.131	−0.19	0.008
IMAT/SAT	0.29	0.0001	0.12	0.217

Hepatic steatosis^b^				
S-ALAT	0.34	1.4 × 10^−9^	0.19	0.0008

Frequent-sampling intravenous glucose tolerance test^c^				
Insulin sensitivity	−0.40	4.3 × 10^−12^	−0.08	0.142
Disposition index	−0.14	0.011	0.02	0.794

*r* = correlation coefficient from a model adjusting for the non-independence among siblings (model 1), and non-independence among siblings and BMI (model 2). VAT/SAT = ratio of visceral to abdominal subcutaneous adipose tissue areas. IMAT/SAT = ratio of thigh intermuscular to thigh subcutaneous adipose tissue areas. S-ALAT = serum alanine aminotransferase.

a *n* = 137.

b *n* = 313.

c *n* = 326.
