# Supplementary material for: CDKN2B expression and subcutaneous adipose tissue expandability: Possible influence of the 9p21 atherosclerosis locus
Source: Biochem Biophys Res Commun. 2014 Apr 18;446(4):1126–31. doi: 10.1016/j.bbrc.2014.03.075 (PMC4003348; doi:10.1016/j.bbrc.2014.03.075)
Supplement: Supplementary data 1 — Supplementary material. [file mmc1.doc]

# *CDKN2B* expression and subcutaneous adipose tissue expandability: possible influence of the 9p21 atherosclerosis locus

Per-Arne Svensson, Björn Wahlstrand, Maja Olsson, Philippe Froguel, Mario Falchi, Richard N. Bergman, Philip G. McTernan, Thomas Hedner, Lena M.S. Carlsson, and Peter Jacobson.

### Tissue distribution of gene expression

To determine the tissue distribution of genes adjacent to the 9p21 locus, DNA microarray expression profiles of 65 human tissues were acquired from the Gene Expression Omnibus (GEO) database (Dataset GSE3526; http://www.ncbi.nlm.nih.gov/geo). The following genes (probeset ID) were investigated: *ANRIL* (1559884_at), *CDKN2A* (207039_at), *CDKN2B* (236313_at), and *MTAP* (211363_s_at). In cases of transcript redundancy, we selected the probeset with the highest sensitivity and specificity according to GeneCards (http://www.genecards.org) and with the highest level of expression. To verify the tissue distribution of *CDKN2B* the Human Total RNA Master Panel II (Clontech Laboratories, Inc., Palo Alto, CA, USA) was used. This RNA panel was supplemented with samples of total RNA prepared from subcutaneous adipose tissue and adipocytes from healthy volunteers (two females, one male with BMI of 22.4 –26.7 kg/m^2^; and two females, one male with BMI of 22.4 –29.3 kg/m^2^, respectively). Adipocytes were isolated as previously described [1].

### Statistical analysis

Correlations among traits, as well as genetic associations between carrier status of risk alleles and gene expression, were analyzed using MIXED procedure in SAS. In the linear mixed models, non-independence among siblings was adjusted for using a “sandwich estimator” of the covariance matrix, which asymptotically yields the same parameter estimates as ordinary least-squares or regression methods, but the standard errors and, consequently, hypothesis tests are adjusted for the dependencies among family members. Allelic associations with *CDKN2B* expression and their possible dependencies upon BMI were analyzed in the Sibpair study, using linear mixed models adjusting for the non-independence among sibs and effects of sex, age, and BMI.

### Identification of adipogenic transcripts correlated with *CDKN2B*

In order to select genes with relevance for adipogenesis, we included genes whose gene ontology biological process (GOBP) annotations (http://www.geneontology.org) included the terms “transcription”, “expression”, “proliferation”, “differentiation”, “angiogenesis”, “endothelial”, or “blood vessel”. Transcript redundancy was eliminated by selecting the most highly expressed probeset of each gene. In the next step, we used the BioGPS database [2] (http://biogps.gnf.org) to estimate the expression of each gene across multiple human tissues, relative to the expression in adipocytes. Using the median as an arbitrary cut-off, the number of genes was reduced further by excluding those whose expression level in adipocytes was below the median of all tissues. These steps resulted in a subset of 942 genes, which was subjected to correlation analysis with *CDKN2B* expression level. Correlation estimates were obtained from linear mixed models adjusting for effects of age, sex, and non-independence among siblings. Using a significance threshold of p=5.3 x 10^-5^ to account for multiple testing, the subset was further reduced to 320 transcripts, which correlated significantly with *CDKN2B* expression. Genes were classified as either promotive or inhibitory based on GOBP annotations or from manual evaluation of the scientific literature. Finally, 61 genes were excluded due to insufficient information about whether their action on proliferation is promotive or inhibitory, resulting in a panel of 259 transcripts (supplementary table II).

### REFERENCES

[1] M. Jernas, J. Palming, K. Sjoholm, E. Jennische, P.A. Svensson, B.G. Gabrielsson, M. Levin, A. Sjogren, M. Rudemo, T.C. Lystig, B. Carlsson, L.M. Carlsson, M. Lonn, Separation of human adipocytes by size: hypertrophic fat cells display distinct gene expression, FASEB journal : official publication of the Federation of American Societies for Experimental Biology 20 (2006) 1540-1542.

[2] C. Wu, C. Orozco, J. Boyer, M. Leglise, J. Goodale, S. Batalov, C.L. Hodge, J. Haase, J. Janes, J.W. Huss, 3rd, A.I. Su, BioGPS: an extensible and customizable portal for querying and organizing gene annotation resources, Genome biology 10 (2009) R130.
