# Supplementary material for: CDKN2B expression and subcutaneous adipose tissue expandability: Possible influence of the 9p21 atherosclerosis locus
Source: Biochem Biophys Res Commun. 2014 Apr 18;446(4):1126–31. doi: 10.1016/j.bbrc.2014.03.075 (PMC4003348; doi:10.1016/j.bbrc.2014.03.075)
Supplement: Supplementary data 2 — Supplementary Figure and Tables. [file mmc2.doc]

Supplement to:

# *CDKN2B* expression and subcutaneous adipose tissue expandability: possible influence of the 9p21 atherosclerosis locus

Per-Arne Svensson, Björn Wahlstrand, Maja Olsson, Philippe Froguel, Mario Falchi, Richard N. Bergman, Philip G. McTernan, Thomas Hedner, Lena M.S. Carlsson, and Peter Jacobson.

Supplementary figure I

Supplementary table I

Supplementary table II

**Supplementary figure I.**

**
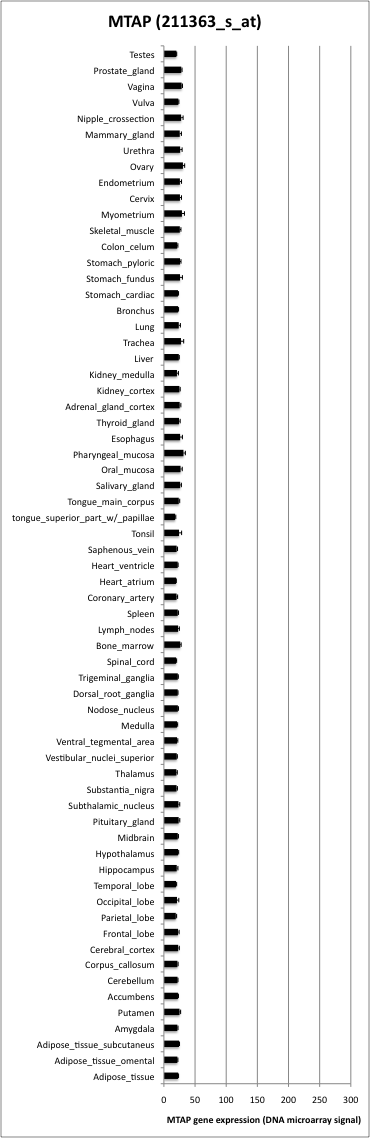

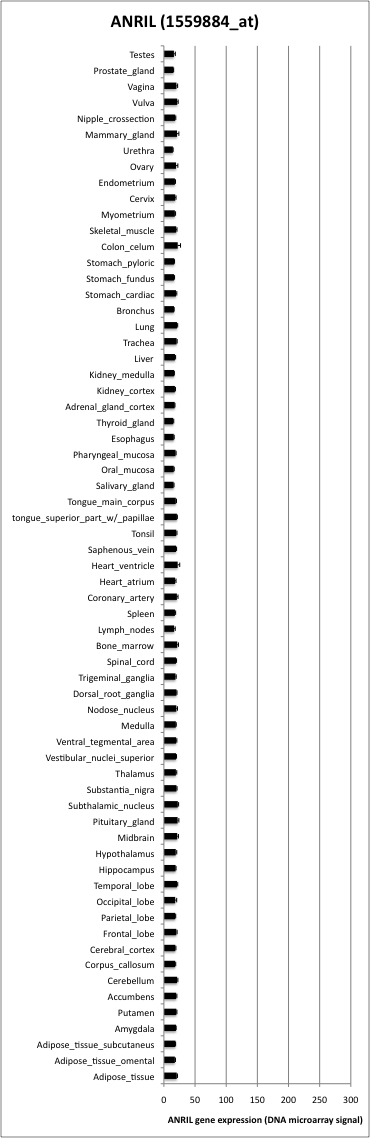
**


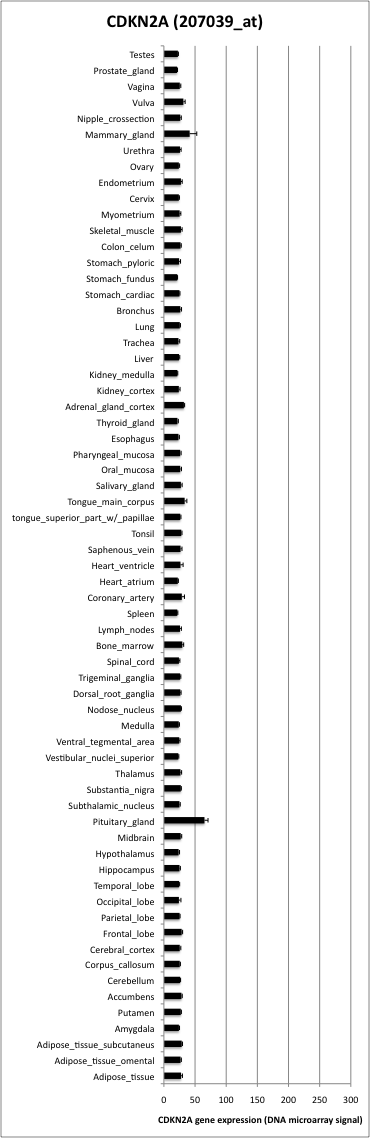

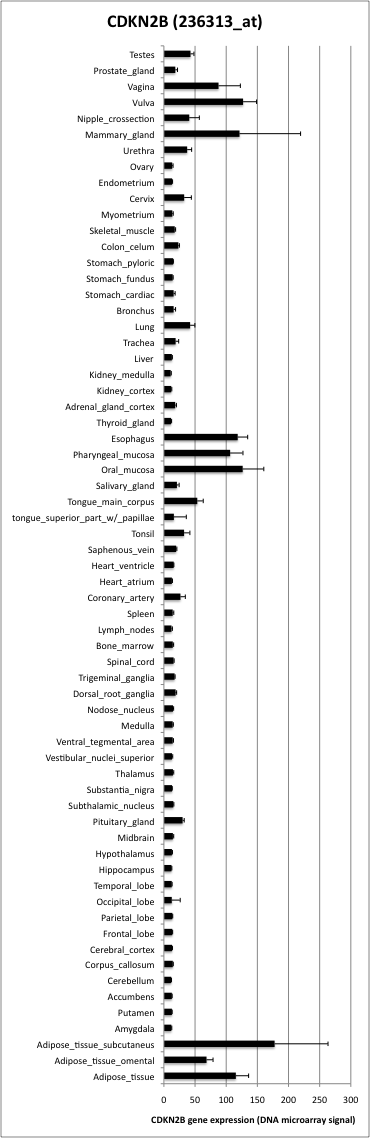


**Supplementary figure I. Expression in 65 human tissues of positional candidate genes at the chromosome 9p21 locus.** DNA microarray signals expressed as mean ± SE. Transcript redundancy was eliminated by considering the probe set with the highest sensitivity and specificity according to GeneCards (http://www.genecards.org) and with the highest level of expression. *ANRIL*, Antisense noncoding RNA in the INK4 locus; *MTAP*, Methylthioadenosine phosphorylase; *CDKN2A*, Cyclin-dependent kinase inhibitor 2A (melanoma, p16, inhibits CDK4); *CDKN2B*, Cyclin-dependent kinase inhibitor 2B (p15, inhibits CDK4).

**Supplementary table I. Covariation between SAT expression of CDKN2B and 259 genes with promotive or inhibitory effects on adipogenesis.**

| **Anti-angiogenic genes positively correlated with CDKN2B** | | |
| --- | --- | --- |
| **Name** | **Symbol** | **Probe set** |
| capping protein (actin filament) muscle Z-line, alpha 2 | CAPZA2 | 201238_s_at |
| caveolin 1, caveolae protein, 22kDa | CAV1 | 212097_at |
| caveolin 2 | CAV2 | 203323_at |
| cystatin C | CST3 | 201360_at |
| endothelin 1 | EDN1 | 222802_at |
| glutamate-cysteine ligase, modifier subunit | GCLM | 203925_at |
| integrin, alpha V (vitronectin receptor, alpha polypeptide, antigen CD51) | ITGAV | 202351_at |
| lysyl oxidase | LOX | 215446_s_at |
| mitogen-activated protein kinase kinase kinase 7 | MAP3K7 | 206854_s_at |
| matrix metallopeptidase 19 | MMP19 | 204575_s_at |
| neurofibromin 1 (neurofibromatosis, von Recklinghausen disease, Watson disease) | NF1 | 212676_at |
| ribonuclease/angiogenin inhibitor 1 | RNH1 | 206050_s_at |
| S100 calcium binding protein A2 | S100A2 | 204268_at |
| transforming growth factor, beta receptor II (70/80kDa) | TGFBR2 | 208944_at |
| thrombospondin 1 | THBS1 | 201108_s_at |
|  |  |  |
| **Anti-angiogenic genes negatively correlated with CDKN2B** | | |
| **Name** | **Symbol** | **Probe set** |
| angiotensinogen (serpin peptidase inhibitor, clade A, member 8) | AGT | 202834_at |
| apolipoprotein E | APOE | 203381_s_at |
| endothelin receptor type A | EDNRA | 204464_s_at |
| natriuretic peptide receptor A/guanylate cyclase A (atrionatriuretic peptide receptor A) | NPR1 | 32625_at |
| phosphatase and tensin homolog (mutated in multiple advanced cancers 1) | PTEN | 225363_at |
| transmembrane protease, serine 6 | TMPRSS6 | 214955_at |
|  |  |  |
| **Pro-angiogenic genes positively correlated with CDKN2B** | | |
| **Name** | **Symbol** | **Probe set** |
| chemokine (C-C motif) ligand 2 | CCL2 | 216598_s_at |
| connective tissue growth factor | CTGF | 209101_at |
| chemokine (C-X-C motif) ligand 12 (stromal cell-derived factor 1) | CXCL12 | 209687_at |
| fibroblast growth factor receptor 1 (fms-related tyrosine kinase 2, Pfeiffer syndrome) | FGFR1 | 211535_s_at |
| hypoxia-inducible factor 1, alpha subunit (basic helix-loop-helix transcription factor) | HIF1A | 200989_at |
| runt-related transcription factor 1 (acute myeloid leukemia 1; aml1 oncogene) | RUNX1 | 209360_s_at |
| serpin peptidase inhibitor, clade E (nexin, plasminogen activator inhibitor type 1), member 1 | SERPINE1 | 202627_s_at |
| SHC (Src homology 2 domain containing) transforming protein 1 | SHC1 | 214853_s_at |
|  |  |  |
| **Pro-angiogenic genes negatively correlated with CDKN2B** | | |
| **Name** | **Symbol** | **Probe set** |
| angiogenin, ribonuclease, RNase A family, 5 | ANG | 205141_at |
| ATP synthase, H+ transporting, mitochondrial F1 complex, beta polypeptide | ATP5B | 201322_at |
| endothelin converting enzyme 1 | ECE1 | 201749_at |
| phosphatidic acid phosphatase type 2B | PPAP2B | 212226_s_at |
| transglutaminase 2 (C polypeptide, protein-glutamine-gamma-glutamyltransferase) | TGM2 | 201042_at |
| vascular endothelial growth factor A | VEGFA | 210512_s_at |
|  |  |  |
| **Anti-transcription genes positively correlated with CDKN2B** | | |
| **Name** | **Symbol** | **Probe set** |
| chromosome 14 open reading frame 156 | C14orf156 | 221434_s_at |
| CCAAT/enhancer binding protein (C/EBP), gamma | CEBPG | 225527_at |
| cAMP responsive element binding protein 3 | CREB3 | 209432_s_at |
| cAMP responsive element binding protein 3-like 2 | CREB3L2 | 212345_s_at |
| E74-like factor 4 (ets domain transcription factor) | ELF4 | 31845_at |
| empty spiracles homeobox 2 | EMX2 | 221950_at |
| engrailed homeobox 1 | EN1 | 220559_at |
| enolase 1, (alpha) | ENO1 | 201231_s_at |
| four and a half LIM domains 2 | FHL2 | 202949_s_at |
| filamin A, alpha (actin binding protein 280) | FLNA | 200859_x_at |
| forkhead box N3 | FOXN3 | 222494_at |
| hexamethylene bis-acetamide inducible 1 | HEXIM1 | 202814_s_at |
| heterogeneous nuclear ribonucleoprotein A/B | HNRNPAB | 201277_s_at |
| homeobox A10 | HOXA10 | 213150_at |
| inhibitor of DNA binding 2, dominant negative helix-loop-helix protein | ID2 | 201565_s_at |
| intraflagellar transport 57 homolog (Chlamydomonas) | IFT57 | 218100_s_at |
| kelch-like ECH-associated protein 1 | KEAP1 | 202417_at |
| LIM and cysteine-rich domains 1 | LMCD1 | 218574_s_at |
| methyl-CpG binding domain protein 2 | MBD2 | 202484_s_at |
| mortality factor 4 like 1 /// mortality factor 4 | MORF4 | 221381_s_at |
| nuclear factor (erythroid-derived 2)-like 1 | NFE2L1 | 200759_x_at |
| nuclear factor (erythroid-derived 2)-like 2 | NFE2L2 | 201146_at |
| nuclear transcription factor Y, beta | NFYB | 218127_at |
| PDZ and LIM domain 1 (elfin) | PDLIM1 | 208690_s_at |
| prolactin regulatory element binding | PREB | 217861_s_at |
| paired related homeobox 1 | PRRX1 | 226695_at |
| polymerase I and transcript release factor | PTRF | 208789_at |
| peptidyl-tRNA hydrolase 2 | PTRH2 | 218732_at |
| retinoblastoma binding protein 8 | RBBP8 | 203344_s_at |
| SMAD family member 3 | SMAD3 | 218284_at |
| signal transducer and activator of transcription 1, 91kDa | STAT1 | 200887_s_at |
| signal transducer and activator of transcription 3 (acute-phase response factor) | STAT3 | 208991_at |
| transcription elongation factor B (SIII), polypeptide 1 (15kDa, elongin C) | TCEB1 | 202824_s_at |
| transducin-like enhancer of split 2 (E(sp1) homolog, Drosophila) | TLE2 | 40837_at |
| vestigial like 3 (Drosophila) | VGLL3 | 227399_at |
| zinc finger E-box binding homeobox 2 | ZEB2 | 203603_s_at |
| zinc fingers and homeoboxes 3 | ZHX3 | 217367_s_at |
| zinc finger protein 219 | ZNF219 | 219314_s_at |
|  |  |  |
| **Anti-transcription genes negatively correlated with CDKN2B** | | |
| **Name** | **Symbol** | **Probe set** |
| chromodomain helicase DNA binding protein 5 | CHD5 | 213965_s_at |
| cAMP responsive element binding protein 3-like 1 | CREB3L1 | 213059_at |
| developmentally regulated GTP binding protein 1 | DRG1 | 202810_at |
| inhibitor of growth family, member 4 | ING4 | 48825_at |
| jumonji domain containing 3, histone lysine demethylase | JMJD3 | 41387_r_at |
| leucine zipper, putative tumor suppressor 1 | LZTS1 | 222107_x_at |
| ribosomal protein S14 /// similar to ribosomal protein S14 | MGC87895 / | 208646_at |
| necdin homolog (mouse) | NDN | 209550_at |
| prohibitin 2 | PHB2 | 201600_at |
| protein kinase (cAMP-dependent, catalytic) inhibitor gamma | PKIG | 202732_at |
| Retinoblastoma binding protein 4 | RBBP4 | 239071_at |
| REST corepressor 1 | RCOR1 | 212612_at |
| ribosomal protein L11 | RPL11 | 200010_at |
| ribosomal protein S14 | RPS14 | 208645_s_at |
| thioredoxin interacting protein | TXNIP | 201010_s_at |
|  |  |  |
| **Pro-transcription genes positively correlated with CDKN2B** | | |
| **Name** | **Symbol** | **Probe set** |
| c-abl oncogene 1, receptor tyrosine kinase | ABL1 | 202123_s_at |
| cAMP responsive element binding protein-like 2 | CREBL2 | 201989_s_at |
| EGFR-coamplified and overexpressed protein | ECOP | 208091_s_at |
| general transcription factor IIH, polypeptide 5 | GTF2H5 | 213357_at |
| interleukin-1 receptor-associated kinase 1 | IRAK1 | 201587_s_at |
| MyoD family inhibitor domain containing | MDFIC | 211675_s_at |
| mediator complex subunit 8 | MED8 | 213127_s_at |
| NIF3 NGG1 interacting factor 3-like 1 (S. pombe) | NIF3L1 | 218133_s_at |
| proteasome (prosome, macropain) 26S subunit, non-ATPase, 9 | PSMD9 | 207805_s_at |
| PYD and CARD domain containing | PYCARD | 221666_s_at |
| arginyl aminopeptidase (aminopeptidase B) | RNPEP | 208270_s_at |
| sequestosome 1 | SQSTM1 | 201471_s_at |
| transcription factor B2, mitochondrial | TFB2M | 218605_at |
| tumor necrosis factor receptor superfamily, member 1A | TNFRSF1A | 207643_s_at |
| Yes-associated protein 1, 65kDa | YAP1 | 224894_at |
|  |  |  |
| **Pro-transcription genes negatively correlated with CDKN2B** | | |
| **Name** | **Symbol** | **Probe set** |
| activin A receptor, type IIB | ACVR2B | 236126_at |
| ash2 (absent, small, or homeotic)-like (Drosophila) | ASH2L | 209517_s_at |
| basic transcription factor 3 | BTF3 | 211939_x_at |
| CDC5 cell division cycle 5-like (S. pombe) | CDC5L | 209056_s_at |
| Cbp/p300-interacting transactivator, with Glu/Asp-rich carboxy-terminal domain, 2 | CITED2 | 209357_at |
| CCHC-type zinc finger, nucleic acid binding protein | CNBP | 206158_s_at |
| ectodysplasin A | EDA | 206217_at |
| estrogen receptor 1 | ESR1 | 205225_at |
| even-skipped homeobox 1 | EVX1 | 207914_x_at |
| HMG-box transcription factor 1 | HBP1 | 209102_s_at |
| H6 family homeobox 1 | HMX1 | 207353_s_at |
| homeobox B2 | HOXB2 | 205453_at |
| homeobox D4 | HOXD4 | 205522_at |
| leucine-rich PPR-motif containing | LRPPRC | 211971_s_at |
| minichromosome maintenance complex component 6 | MCM6 | 201930_at |
| mediator complex subunit 16 | MED16 | 43544_at |
| MAX-like protein X | MLX | 213708_s_at |
| MLX interacting protein-like | MLXIPL | 221163_s_at |
| nucleophosmin (nucleolar phosphoprotein B23, numatrin) | NPM1 | 200063_s_at |
| nuclear receptor subfamily 3, group C, member 1 (glucocorticoid receptor) | NR3C1 | 201865_x_at |
| nuclear receptor interacting protein 1 | NRIP1 | 202600_s_at |
| pterin-4 alpha-carbinolamine dehydratase/dimerization cofactor of hepatocyte nuclear factor 1 alpha | PCBD1 | 203557_s_at |
| paired-like homeodomain 3 | PITX3 | 208277_at |
| pleiomorphic adenoma gene-like 1 | PLAGL1 | 209318_x_at |
| retinoic acid receptor, gamma | RARG | 204189_at |
| ribosomal protein L6 | RPL6 | 200034_s_at |
| ribosomal protein S16 | RPS16 | 226131_s_at |
| sirtuin (silent mating type information regulation 2 homolog) 3 (S. cerevisiae) | SIRT3 | 49327_at |
| ubiquitin A-52 residue ribosomal protein fusion product 1 | UBA52 | 221700_s_at |
|  |  |  |
| **Anti-expression genes positively correlated with CDKN2B** | | |
| **Name** | **Symbol** | **Probe set** |
| - | - | - |
|  | | |
| **Anti-expression genes negatively correlated with CDKN2B** | | |
| **Name** | **Symbol** | **Probe set** |
| BCL2/adenovirus E1B 19kDa interacting protein 3 | BNIP3 | 201849_at |
|  |  |  |
| **Pro-expression genes positively correlated with CDKN2B** | | |
| **Name** | **Symbol** | **Probe set** |
| - | - | - |
|  |  |  |
| **Pro-expression genes negatively correlated with CDKN2B** | | |
| **Name** | **Symbol** | **Probe set** |
| - | - | - |
|  |  |  |
| **Anti-proliferative genes positively correlated with CDKN2B** | | |
| **Name** | **Symbol** | **Probe set** |
| ADAM metallopeptidase with thrombospondin type 1 motif, 1 | ADAMTS1 | 222162_s_at |
| annexin A1 | ANXA1 | 201012_at |
| annexin A7 | ANXA7 | 201366_at |
| beclin 1, autophagy related | BECN1 | 208946_s_at |
| cyclin-dependent kinase inhibitor 1A (p21, Cip1) | CDKN1A | 202284_s_at |
| chemokine-like factor | CKLF | 221058_s_at |
| CCR4-NOT transcription complex, subunit 8 | CNOT8 | 202164_s_at |
| cellular repressor of E1A-stimulated genes 1 | CREG1 | 201200_at |
| catenin (cadherin-associated protein), alpha 1, 102kDa | CTNNA1 | 210844_x_at |
| deleted in liver cancer 1 | DLC1 | 210762_s_at |
| elastin (supravalvular aortic stenosis, Williams-Beuren syndrome) | ELN | 212670_at |
| epithelial membrane protein 1 | EMP1 | 201324_at |
| epithelial membrane protein 3 | EMP3 | 203729_at |
| epidermal growth factor receptor pathway substrate 8 | EPS8 | 202609_at |
| ecotropic viral integration site 5 | EVI5 | 209717_at |
| coagulation factor II (thrombin) receptor | F2R | 203989_x_at |
| ferritin, heavy polypeptide 1 | FTH1 | 200748_s_at |
| glycoprotein (transmembrane) nmb | GPNMB | 201141_at |
| RAP1B, member of RAS oncogene family /// hCG1757335 | hCG_175733 | 200833_s_at |
| heme oxygenase (decycling) 1 | HMOX1 | 203665_at |
| interferon, gamma-inducible protein 16 | IFI16 | 206332_s_at |
| insulin induced gene 1 | INSIG1 | 201626_at |
| leucine zipper, down-regulated in cancer 1 | LDOC1 | 204454_at |
| hypothetical LOC729659 | LOC729659 | 208540_x_at |
| leukocyte specific transcript 1 | LST1 | 214574_x_at |
| microtubule-associated protein, RP/EB family, member 1 | MAPRE1 | 200713_s_at |
| hypothetical protein MGC5618 | MGC5618 | 221477_s_at |
| msh homeobox 1 | MSX1 | 205932_s_at |
| Notch homolog 2 (Drosophila) | NOTCH2 | 202443_x_at |
| phosphatidylethanolamine N-methyltransferase | PEMT | 207621_s_at |
| phosphatidic acid phosphatase type 2A | PPAP2A | 210946_at |
| proteoglycan 4 | PRG4 | 206007_at |
| quiescin Q6 sulfhydryl oxidase 1 | QSOX1 | 201482_at |
| retinol dehydrogenase 11 (all-trans/9-cis/11-cis) | RDH11 | 217776_at |
| S100 calcium binding protein A11 | S100A11 | 200660_at |
| S100 calcium binding protein A6 | S100A6 | 217728_at |
| stratifin | SFN | 33322_i_at |
| S-phase kinase-associated protein 2 (p45) | SKP2 | 203625_x_at |
| superoxide dismutase 2, mitochondrial | SOD2 | 215223_s_at |
| transcription factor Dp-1 | TFDP1 | 212330_at |
| transforming growth factor, beta 1 | TGFB1 | 203085_s_at |
| transforming growth factor, beta-induced, 68kDa | TGFBI | 201506_at |
| transforming growth factor, beta receptor III | TGFBR3 | 226625_at |
| tumor protein p53 | TP53 | 201746_at |
| taxilin alpha | TXLNA | 212300_at |
| ubiquitin carboxyl-terminal esterase L1 (ubiquitin thiolesterase) | UCHL1 | 201387_s_at |
| zinc finger E-box binding homeobox 1 | ZEB1 | 212764_at |
|  |  |  |
| **Anti-proliferative genes negatively correlated with CDKN2B** | | |
| **Name** | **Symbol** | **Probe set** |
| ADAM metallopeptidase with thrombospondin type 1 motif, 8 | ADAMTS8 | 235649_at |
| alpha-2-glycoprotein 1, zinc-binding | AZGP1 | 209309_at |
| alpha-2-glycoprotein 1, zinc-binding /// similar to ZN-alpha-2-glycoprotein | AZGP1 /// | 217014_s_at |
| cyclin-dependent kinase inhibitor 1C (p57, Kip2) | CDKN1C | 213348_at |
| cyclin-dependent kinase inhibitor 2C (p18, inhibits CDK4) | CDKN2C | 204159_at |
| cyclin-dependent kinase inhibitor 2D (p19, inhibits CDK4) | CDKN2D | 210240_s_at |
| nuclear factor of kappa light polypeptide gene enhancer in B-cells inhibitor-like 1 | NFKBIL1 | 209973_at |
| tensin like C1 domain containing phosphatase (tensin 2) | TENC1 | 212494_at |
| tumor necrosis factor receptor superfamily, member 13B | TNFRSF13B | 207641_at |
|  |  |  |
| **Pro-proliferative genes positively correlated with CDKN2B** | | |
| **Name** | **Symbol** | **Probe set** |
| ADAM metallopeptidase domain 17 (tumor necrosis factor, alpha, converting enzyme) | ADAM17 | 205745_x_at |
| adrenomedullin | ADM | 202912_at |
| adrenergic, alpha-2A-, receptor | ADRA2A | 209869_at |
| branched chain aminotransferase 1, cytosolic | BCAT1 | 225285_at |
| cyclin D2 | CCND2 | 200953_s_at |
| CD86 molecule | CD86 | 210895_s_at |
| cell division cycle 123 homolog (S. cerevisiae) | CDC123 | 201725_at |
| clusterin | CLU | 208792_s_at |
| cold shock domain containing E1, RNA-binding | CSDE1 | 202646_s_at |
| chemokine (C-X-C motif) ligand 10 | CXCL10 | 204533_at |
| discoidin domain receptor tyrosine kinase 2 | DDR2 | 225442_at |
| guanine nucleotide binding protein (G protein), beta polypeptide 1 | GNB1 | 200746_s_at |
| granulin | GRN | 200678_x_at |
| isoprenylcysteine carboxyl methyltransferase | ICMT | 201609_x_at |
| NCK adaptor protein 1 | NCK1 | 211063_s_at |
| platelet derived growth factor D | PDGFD | 219304_s_at |
| ras-related C3 botulinum toxin substrate 2 (rho family, small GTP binding protein Rac2) | RAC2 | 213603_s_at |
| signal sequence receptor, alpha (translocon-associated protein alpha) | SSR1 | 200891_s_at |
| TBC1 domain family, member 8 (with GRAM domain) | TBC1D8 | 204526_s_at |
| TIMP metallopeptidase inhibitor 1 | TIMP1 | 201666_at |
| thioredoxin | TXN | 208864_s_at |
|  |  |  |
| **Pro-proliferative genes negatively correlated with CDKN2B** | | |
| **Name** | **Symbol** | **Probe set** |
| acetylcholinesterase (Yt blood group) | ACHE | 205377_s_at |
| angiotensin II receptor, type 1 | AGTR1 | 205357_s_at |
| CD70 molecule | CD70 | 206508_at |
| cholinergic receptor, nicotinic, beta 2 (neuronal) | CHRNB2 | 206635_at |
| COP9 constitutive photomorphogenic homolog subunit 2 (Arabidopsis) | COPS2 | 202467_s_at |
| fibroblast growth factor 4 (heparin secretory transforming protein 1, Kaposi sarcoma oncogene) | FGF4 | 206783_at |
| insulin receptor substrate 1 | IRS1 | 204686_at |
| insulin receptor substrate 2 | IRS2 | 209185_s_at |
| ribosomal protein S4, X-linked /// similar to Ribosomal protein S4, X-linked /// hypothetical LOC442162 | LOC1001281 | 216342_x_at |
| NADH dehydrogenase (ubiquinone) Fe-S protein 4, 18kDa (NADH-coenzyme Q reductase) | NDUFS4 | 209303_at |
| nuclear receptor subfamily 6, group A, member 1 | NR6A1 | 227494_at |
| nardilysin (N-arginine dibasic convertase) | NRD1 | 208709_s_at |
| ribosomal protein S15a | RPS15A | 200781_s_at |
| ribosomal protein S4, X-linked | RPS4X | 200933_x_at |
| signal transducer and activator of transcription 5A | STAT5A | 203010_at |
|  |  |  |
| **Anti-differentiation genes positively correlated with CDKN2B** | | |
| **Name** | **Symbol** | **Probe set** |
| ADAM metallopeptidase domain 9 (meltrin gamma) | ADAM9 | 202381_at |
| cyclin D1 | CCND1 | 208712_at |
| chordin-like 1 | CHRDL1 | 209763_at |
| cystatin A (stefin A) | CSTA | 204971_at |
| endothelin receptor type B | EDNRB | 204271_s_at |
| exostoses (multiple) 2 | EXT2 | 202012_s_at |
| four and a half LIM domains 1 | FHL1 | 201540_at |
| growth arrest-specific 7 | GAS7 | 202192_s_at |
| glutathione peroxidase 1 | GPX1 | 200736_s_at |
| integrin, beta 1 (fibronectin receptor, beta polypeptide, antigen CD29 includes MDF2, MSK12) | ITGB1 | 211945_s_at |
| leptin | LEP | 207092_at |
| muscleblind-like (Drosophila) | MBNL1 | 201152_s_at |
| serpin peptidase inhibitor, clade E (nexin, plasminogen activator inhibitor type 1), member 2 | SERPINE2 | 212190_at |
| slit homolog 2 (Drosophila) | SLIT2 | 209897_s_at |
| ubiquitin-conjugating enzyme E2 variant 1 /// TMEM189-UBE2V1 | TMEM189-UB | 201002_s_at |
| WW domain containing transcription regulator 1 | WWTR1 | 202133_at |
|  |  |  |
| **Anti-differentiation genes negatively correlated with CDKN2B** | | |
| **Name** | **Symbol** | **Probe set** |
| - | - | - |
|  | | |
| **Pro-differentiation genes positively correlated with CDKN2B** | | |
| **Name** | **Symbol** | **Probe set** |
| disabled homolog 2, mitogen-responsive phosphoprotein (Drosophila) | DAB2 | 201280_s_at |
| guanine nucleotide binding protein (G protein) alpha 12 | GNA12 | 224681_at |
| inhibin, beta B | INHBB | 205258_at |
| lectin, galactoside-binding, soluble, 1 (galectin 1) | LGALS1 | 201105_at |
| neuronal cell adhesion molecule | NRCAM | 204105_s_at |
| secreted frizzled-related protein 4 | SFRP4 | 204051_s_at |
| suppressor of cytokine signaling 5 | SOCS5 | 208127_s_at |
|  |  |  |
| **Pro-differentiation genes negatively correlated with CDKN2B** | | |
| **Name** | **Symbol** | **Probe set** |
| acyl-Coenzyme A dehydrogenase, C-4 to C-12 straight chain | ACADM | 202502_at |
| activin A receptor, type IIA | ACVR2A | 228416_at |
| calcium/calmodulin-dependent protein kinase I | CAMK1 | 204392_at |
| ELAV (embryonic lethal, abnormal vision, Drosophila)-like 3 (Hu antigen C) | ELAVL3 | 206338_at |
| fragile X mental retardation, autosomal homolog 1 | FXR1 | 201637_s_at |
| insulin-like growth factor binding protein 3 | IGFBP3 | 210095_s_at |
| lectin, galactoside-binding, soluble, 3 | LGALS3 | 208949_s_at |
| lipin 1 | LPIN1 | 212276_at |
| NK2 transcription factor related, locus 5 (Drosophila) | NKX2-5 | 206578_at |
| paired box 5 | PAX5 | 206802_at |
| peroxisome proliferator-activated receptor gamma | PPARG | 208510_s_at |

Gene expression in SAT as measured by DNA microarray in the Sibpair study. Correlations analyzed by mixed models adjusting for effects of age, sex, and non-independence among siblings. Genes were selected as described in the Methods section.

**Supplementary table II. Characteristics of the human subjects in this study.**

|  | Sibpair study | |  | Depot study | |  | BB study |
| --- | --- | --- | --- | --- | --- | --- | --- |
|  | Lean | Obese |  | Lean | Obese |  | Obese |
| n | 171 | 183 |  | 5 | 5 |  | 10 |
| Sex (M/F) | 54/117 | 52/131 |  | 0/5 | 0/5 |  | 5/5 |
| Age (yr) | 36.7 ± 8.0 | 37.9 ± 7.2 |  | 40 ±7 | 50 ± 4 |  | 41.4 ± 10.3 |
| BMI (kg/m^2^) | 22.6 ± 2.1 | 36.3 ± 5.9 |  | 23.0 ± 1.2 | 33.2 ± 3.1 |  | 38.8 ± 5.1 |

Values are presented as counts or mean ± SD.
